# The Transfusion of Blood*Read before the Surgical Section of the Buffalo Academy of Medicine, October 7, 1914.

**Published:** 1915-01

**Authors:** Alfred H. Noehren

**Affiliations:** Buffalo, N. Y.


### The Transfusion of Blood.*

ALFRED H. NOEHREN, M. D.,

Buffalo, N. Y.

The subject of the transfusion of blood is not a new one. It dates back almost to the discovery of the circulation by Harvey in 1628. It was shortly after this that Christopher Wren first conceived the idea of infusion into the blood and experimented on dogs by infusing solutions of opium into their veins. The first infusion on a human being was done in 1657 by de Bordeaux on a criminal sentenced to be hung. The first transfusions were done on dogs by Richard Lower in 1666, by means of a silver canula passing from the common carotid artery of the one to the internal jugular vein of the other. They were very successful. Lower did his first transfusion on a human being late in 1667, using the blood of a lamb, with good results.

However, a few months prior to this, i.e., early in 1667, in the city of Paris, Jean Denis had already done a human transfusion, the first that had ever been done. This first transfusion was done on a fever patient who had been almost exsanguinated by the then prevalent method of severe bleeding in all cases of sickness. Lamb's blood was used and the sudden restoration of the patient to health was quite remarkable. Denis' second case was equally successful, but the third died shortly after the operation from arsenic surreptitiously administered to the patient by his wife. This death was used by the jealous colleagues of Denis to discredit the operation and to the passage of laws in France forbidding transfusion.

These transfusions in France and England were followed by others in Italy, Germany, and Holland, but the procedure was soon dropped, probably on account of the frequent failures or on account of opposition for other reasons, as in France. Nothing further is heard of transfusion until the beginning of the 19th century.

Up to this time the canula method had been used exclusively. Early in the 19th century Blundell first used the syringe method. Dumas and Provost in 1821 showed the injurious effect of transfusing blood from one species into another and the possibility of using defibrinated blood. Dieffenbach, Bischoff, Magendie, and Brown-Sequard were further investigators at this time and all used defibrinated blood, usually from man to man. In 1864, Traube first used transfusion in Coal Gas Poisoning, whereas previous to this it had been used only in the various anemias, and a few years later Landois and

*Read before the Surgical Section of the Buffalo Academy of Medicine, October 7, 1914.

Eulenburg used it as a routine in acute poisonings of various kinds, such as caused by Coal Gas, Chloroform, Ether, Strychnine, Opium and Phosphorus, in each of these cases preceding the transfusion by bleeding of the patient.

In 1875, Leonard Landois, in his excellent book, "Die Transfusion Des Blutes," from which the above historical facts have been gleaned, collected all the transfusions reported up to that time. Those transfused with human blood were as follows:

| | No of Cases | | |
|---|---|---|---|
| | Cured. | Not Cured. | Doubtful. |
| For traumatic hemorrhage ....... | 12 | 1 | 9 |
| For Post-Partum Hemorrhage.... | 63 | 39 | 4 |
| For other Uterine Hemorrhage and that caused by Neoplasms..... | 8 | 6 | 13 |
| For Intestinal Hemorrhage ...... | 10 | 8 | |
| For Hemorrhage due to Hemophilia | 8 | 11 | 1 |
| For various Anemias and Chlorosis | 20 | 18 | 1 |
| For Asphyxia ................. | 1 | 5 | |
| For Coal Gas Poisoning.......... | 6 | 8 | 1 |
| For Phosphorus Poisoning ...... | 1 | | |
| For Uraemia ................... | 1 | | |
| For Hydrophobia .............. | | 1 | |
| For Septicemia and Pyaemia, including Puerperal Sepsis....... | 4 | 24 | 1 |
| Miscellaneous Cases ............ | 16 | 51 | 1 |
| Total ........................ | 150 | 180 | 12 |

Total, 342 cases.

Soon after this, with the more general use of intravenous infusion of saline solution and with the realization of the dangers of intravascular clotting when defibrinated blood was used, transfusion was again gradually abandoned. In 1883, von Bergman reviewed the whole subject and said the only allowable transfusion was direct from artery to vein. At that time no method had been devised to accomplish this.

However, in the last fifteen years, transfusion has been resurrected and with the greatly improved and simplified technique and newer methods of eliminating its dangers, gives promise of being one of our most valuable therapeutic agencies.

**Indications for Transfusion.** Transfusion is a specific in Acute Hemorrhage from any cause, such as following a ruptured ectopic operation, post-partum or traumatic hemorrhages. In these cases a direct transfusion after the bleeding has been controlled, or where this is impossible, even before it is controlled, not only saves life, but greatly shortens an otherwise long and dangerous convalescence. In cases that are still bleeding, small quantities must be given at a time, so as not to raise the blood-pressure too much, but in cases where the bleeding has ceased, the vessels should be well filled. The improvement in these cases is remarkable.

For the prevention and relief of surgical shock and to prepare anemic cases for severe operation, transfusion is of great value. Crile and Soresi advise having the donor attached to the patient during the operation and intermittently allowing the blood to flow as needed. This is the ideal method. Many cases have been reported of patients that would surely have succumbed to a necessary dangerous operation brought into temporary condition by a transfusion shortly before the operation. The writer has personally observed two cases of splenectomy performed on very anemic patients after a transfusion of blood from their wives. Cullen goes so far as to say that "Transfusion will certainly in the near future become a routine procedure in cases in which operations are required on patients with a very low hemoglobin."

A third very definite indication for transfusion is in cases of Hemorrhagic Diseases of the New-Born. Lespinasse has recently reported 37 cases from the literature including 14 of his own, with 34 recoveries, the 3 that died having been syphilitics. He concludes as follows: "Direct transfusion of blood stops the bleeding, restores the lost blood, and aids in overcoming infection. Direct transfusion has cured where all other methods have failed. It should be used early, but so long as there is a spark of life left, it is never too late to transfuse."

In hemorrhage from Hemophilia or Prolonged Jaundice, transfusion is also of positive benefit.

In the aplastic anemias, especially Pernicious Anemia, in Splenic Anemia, Leukaemia, and Hodgkin's Disease, transfusion is of doubtful value and further experience alone will tell whether it is of any value at all. In Pernicious Anemia many writers, including Crile, claim that there is no benefit. On the other hand, others with equal experience claim at least a temporary benefit. Ottenberg, who has observed 12 cases of pernicious anemia that were transfused, in a personal communication to the writer, says, "My experience in pernicious anemia transfusions on the whole is good, in fact, I have a much better impression than the published literature gives and do not hesitate to recommend it in any case. It does not cure, but it often gives marked relief extending over long periods of time, sometimes as long as two years." Bernheim has seen temporary improvement, but believes better results will be obtained if transfused early, as so far no case has been reported in which the hemoglobin was 50% or more. He also advises frequent transfusions of small amounts. Ellsworth Eliot and Floercken each report a case of marked improvement. The writer's case, which is reported below, showed no improvement. In splenic anemia, transfusion is of chief value in preparing the patient for a splenectomy.

Pellagra is another disease in which transfusion has given good results.

In certain drug poisonings, gas poisonings, and uraemia, transfusion may be of benefit, provided the central nervous system has not already been damaged too much. It should be preceded by bleeding of the patient.

In Typhoid, Tuberculosis, various Septic conditions, and the Toxaemia of Pregnancy the results are encouraging. In Typhoid an immune donor is preferable and in Toxaemia of Pregnancy the donor should be a healthy pregnant woman.

Transfusion has also been suggested in certain chronic skin disorders, as Pemphigus and Psoriasis, and even in Chronic Infections and cases of lowered vitality that do not respond to the usual forms of treatment.

In general, as shown by Soresi, transfusion is the only therapeutic agent for those cases in which the hemopoietic organs are impaired to such an extent that death is inevitable unless the morphological elements and active principles generally furnished by these organs are supplied artificially to keep the body alive until these organs recuperate. Where these organs are still able to supply these elements rapidly, a simple infusion of saline may be sufficient, but when they cannot do this rapidly, the infusion is not sufficient to sustain life. And in conditions in which hemorrhage has not yet been controlled, blood is preferable to salt solution on account of its hemostatic effect.

**Dangers of Transfusion.** The two chief dangers of transfusion, hemolysis and agglutination, can be positively excluded by careful preliminary blood tests. Ottenberg and Kaliski were able to prevent accidents from this cause in a series of 125 transfusions. When there is no hemolysis or agglutination between the bloods of the donor and the patient in the test-tube, there never is in the body of the patient, and where the test-tube shows slight hemolysis or agglutination, there may be no symptoms of bad effects on transfusion, although they probably do occur in a slight degree.

Hemolysis is very common in Tuberculosis and is almost a diagnostic test. In these cases, as well as in others, especially the aplastic anemias, it is sometimes difficult to find a donor whose blood is compatible with the patient, but in the above series, Ottenberg and Kaliski were unable to find donors in only two cases. When hemolysis occurs, it manifests itself by a chill, dyspnoea, hemoglobinuria, and very rarely in severe cases, by death.

Agglutination is not a positive contra-indication, but, if possible, should be avoided. Agglutination of the patient's cells by the donor's serum is less dangerous than the reverse, because a greatly diluted serum is distributed over a large number of cells. However, where there is no great haste, there is no excuse for either of these phenomena to occur, as they can be prevented by a test requiring only three hours time and, according to Ottenberg's method, as much blood as can be secured from a finger-prick of both patient and donor. Where great haste is necessary, it is perfectly justifiable to take a chance, as hemolysis and agglutination sufficiently strong to be dangerous to life are exceedingly rare when the blood is taken from a healthy individual. It is a little less common between blood-relatives.

Another danger of transfusion is the transmission of disease from the donor to the patient. A physical examination of the donor should always be made, and, as the chief danger is from syphilis, a Wassermann should be done at the same time that the hemolysis tests are made.

As to the danger of collapse of the donor, this can easily be prevented by not taking more than one-fourth of his entire blood. This can be estimated from his body-weight. Usually much less than this is needed and in exceptional cases two or more donors may be used for one transfusion.

There remains the danger of overwhelming the already weak and anemic heart of the patient by a sudden rise in blood pressure. By transfusing slowly, especially in the beginning, until the anemic heart muscle recuperates, this danger too can usually be avoided. It certainly is no more dangerous than giving the patient an intra-venous injection of salt solution.

**Methods of Transfusion.** The many methods of transfusion that have been devised, especially in the last fifteen years, may be divided into three groups: 1. those that bring the intima of the donor's blood-vessel directly in contact with that of the recipient, 2. those in which the vessel of the donor is joined to the vessel of the recipient by an intervening tube, 3. those in which the blood of the donor is drawn into some kind of container and then injected into the recipient.

In the **first** group, the suture method of Carrel was first used. This was soon replaced by the now well-known Crile canula, and even today, with its many modifications, this is the most commonly used method. It consists of a small silver ring with several grooves on its surface and a small handle projecting from one edge. The method consists in drawing the cut end of the artery of the donor, usually the radial, through the ring and after turning it back over the ring like a cuff, by means of three guy sutures, tieing it in place. The cut end of the vein of the recipient, usually a vein of the arm, is then drawn over this cuff by similar guy sutures and tied in place, thus bringing the intima of one vessel in direct contact with the intima of the other without any intervening foreign substance. The temporary clamps that had been placed on the vessels are then removed and the blood allowed to flow.

As stated above, a great many modifications of this method have been devised. In 1906, it was the privilege of the writer to assist Ottenberg, while fellow-internes together, in a series of animal experiments on blood-vessel anastomosis and later in several transfusions on human beings. Ottenberg used a ring very much like Crile's, except that it had no handle and was held by a specially-made forceps. He had worked out this method independently without knowing that Crile and others were already using an almost exactly similar one.

A few of the other modifications of this method with their authors are as follows:

Bernheim—Modified Crile canula with prongs.

Hennington—Modified Crile canula with hooks.

Landon—Similar to Hennington.

McGrath—Canula on each half of forceps, vessels attached to hooks, and halves of forceps then brought together.

Soresi—Two canulas sliding on a bar. Vessels cuffed over each and canulas brought together. Soresi advises transfusing into the external jugular vein as being the most direct route to the heart.

Elsberg—Canula that can be adjusted to different sizes by separating its halves and hooks used in place of guy sutures.

Janeway—Two canulas similarly adjustable by means of spring handles and fitting into each other.

In most of these methods artery is joined to vein, in some of them, vein to vein, the greater pressure in the donor causing the blood to flow into the patient.

While theoretically the bringing of intima in direct contact with intima is the ideal method, the technique is so difficult and the failures so frequent that it is impractical for general use. The difficulties can hardly be imagined by one who has not tried this method and no one ought to attempt such a transfusion without previous experience on animals. Further disadvantages of this method are that the donor must be brought into close contact with the patient, that it requires the marking of both with a fairly large scar, and that there is no exact and easy method of measuring the amount of blood passing from one to the other.

In the **second** group in which the vessels of donor and recipient are joined by an intervening tube, the technique is simplified to a certain extent. These tubes may be of glass, as devised by Brewer, or of rubber with glass tips as used by Pope. These tubes are coated with paraffine and pass from artery to vein. Except for the simplified technique, the disadvantages are the same as in the first group and in addition the contact of the blood with a foreign body.

In the **third** group the blood is drawn into a container, usually a syringe, and then injected into the patient. David and Curtis use a glass bulb with a capacity of 400 c.c. with two canula tips blown into one end and a syringe attached to the other. The canula tips fit into the veins of donor and recipient and the blood is alternately aspirated into the bulb from the donor and injected into the recipient. Satterlee and Hooker insert a canula into both donor and recipient, then through an air-filter suck up the blood fron one into a pipette and blow it into the other. Kimpton and Brown use specially formed glass cylinders, which are inserted into the donor's artery and allowed to fill and then forced into the recipient's vein with a cautery bulb pump. Crotti uses a syringe with a blunt needle. Aveling uses an instrument very much like a Davidson syringe with glass tips at each end. Cooley and Vaughan report a case in which they used a simple 10 c.c. hypodermic syringe without the use of any anti-coagulant and recently McGrath advised the same method. In all the others mentioned, paraffine was used to coat the receptacles. Satterlee and Hooker have done some transfusions by using Herudin as an anti-coagulant. Crotti has shown that blood in a dry sterile receptacle clots in 5-8 minutes, but if the receptacle is boiled in salt solution or albolene, the blood clots in 7-12 minutes.

In most of these methods incisions must be made into donor and patient, the blood remains outside of the body for some time, a paraffine coating must be applied, which alone is not an easy procedure, and the technique is more or less complicated. Doubtless any one of them is good when the operator has sufficiently mastered the details and practiced the method.

There remains the method described by Lindeman which seemed to the writer the most practical of all, and after observing several transfusions done by Lindeman and others by this method, decided to use it on a case that recently presented itself. The method will be described in connection with this case.

Mrs. O., age 62, previous history negative except for obscure abdominal pain for a number of years. February, 1912, began to feel run down and lose appetite. Was not seen again until July, 1913, when felt weak and dizzy, looked very pale, and had a pulse of 100. A blood-count done shortly afterward showed 6000 leucocytes, 1,250,000 R.B.C., 60% Hemoglobin, moderate poikilocytosis, a considerable number of microcytes, and a few macrocytes. A probable diagnosis of Pernicious Anemia was made, which became positive as time went on. All the usual remedies were tried, the patient improving and then growing worse again. She developed a severe neuritis of the arm, from which she finally recovered. In March, 1914, went to Toronto, where became rapidly worse. A blood-count done by Dr. Mosier of Toronto showed 28% Hemoglobin and 1,000,000 R.B.C. She was taken home to Buffalo and again seen by the writer. Pulse 120, mental condition confused, dyspnoic, with a corpse-like color. She grew worse for the next few days and her death was expected daily. Nevertheless she rallied and improved gradually to such an extent that she could be up and about and even sing and accompany herself on the piano. Her hemoglobin went up to 75%, but her R.B.C. did not exceed 1,280,000.

In June, 1914, she began to fail again and rapidly went down hill. On July 17, her Hemoglobin was down to 30% and she was failing rapidly and several times death seemed imminent. A transfusion was suggested as something that might be tried in an otherwise hopeless condition, and her husband, after studying the subject thoroughly, asked that it be done. She was taken to the German Deaconess Hospital on July 22, where the writer, with the co-operation of Dr. Joseph Lewis, transfused her by the Lindeman method.

A strong healthy donor was easily found. Blood tests were made by Dr. John Eckel and pronounced negative for hemolysis and agglutination. A Wasserman test of the donor's blood was also negative.

**Operation.** Donor and recipient were placed on tables about 5 feet apart with the instrument table joining their heads. Eight special 20 c.c. Record syringes were sterilized in alcohol and rinsed in sterile water and salt solution. Two sets of Lindeman canulas were sterilized in the same way and coated with Liquid Albolene. Each set consists of three canulas telescoped within each other, the innermost one being a hollow needle and projecting beyond the middle one, which in turn projects beyond the outer one. The middle and outer ones have blunt ends, while the opposite end of the outer one fits on the tip of a Record syringe.

The skin of the adjacent arms of donor and recipient were sterilized with Iodine and Alcohol and rubber tube tourniquets applied. Attempts were then made to introduce a canula into a vein of the patient, but as her veins were barely visible and looked like threads, this was abandoned after several unsuccessful attempts. A ½-in. incision was then made under cocaine over the radial vein at the wrist, when the canula was easily introduced, the inner and middle canula being withdrawn as soon as the outer canula had entered the vein, thus causing no injury to the intima. A syringe with salt solution was at once attached, a small quantity injected to wash out any blood from the canula, and the syringe held in place to prevent bleeding. The tourniquet was then removed.

Another canula was easily introduced directly into the median basilic vein of the donor, an empty syringe attached and slowly filled with blood. In this case the tourniquet was not removed. As soon as the syringe was filled, it was passed over to the operator stationed at the patient and injected through the canula, another syringe being meanwhile filled at the donor. When a syringe had been emptied into the recipient, it was passed to the nurse, who cleaned it by rinsing it in two basins of sterile water and one of salt solution and placed it beside the empty syringes ready to be used again. Small amounts of salt solution were from time to time injected into the patient between the syringes of blood to prevent any possible coagulation of blood in the canula. In this way 10 syringes full of blood, or 200 c.c., were injected into the patient.

The donor showed no bad effects whatever. After resting on the table for a short time, he got up, dressed, went downstairs and cranked an automobile. As for the patient, the immediate effect was good. From being apathetic and listless before the operation, she became bright and talkative and seemed to fell better than she had for a long time. Her color also improved markedly. But in a few hours this condition passed away and she was no better than before the transfusion, and although the interne reported a blood-count the next day of 3,000,000 R.B.C. and 50% Hemoglobin, she kept on going down hill and died 11 days after the operation.

The method of transfusion, however, proved to be a most practical one and to the writer seems the best that has been offered thus far. The blood remains outside of the body for so short a time, 5-15 seconds, that there is no danger of any change in the blood. The amount of blood transfused can be measured easily and accurately. In most cases no incision is necessary and never in the donor, making it easier to procure a donor. The same vein may be used repeatedly. There is no contact of donor and patient and a screen may even be placed between the two.

Although inattention to details may make any method impractical, if done with care, this method of transfusion is simple and easy. Lindeman, in April, 1913, had done 36 transfusions by this method without a single failure or mishap. As recently as August, 1912, the editor of the Journal of the American Medical Association wrote as follows: "It would often be desirable to transfuse ——— if the technique were as simple as, for instance, the intravenous injection of salt solution." Such a technique has now been found, and where a transfusion of blood is really indicated, there is no longer any excuse for leaving it undone.

Note—Since reading this paper, the writer has done two additional transfusions with Dr. Burt C. Johnson. Both were successful, both as to technique and as to benefitting the patient. Some of the syringes worked hard after awhile, which was found to be due to the expansion of the metal plungers by the warm solutions. This can easily be prevented by keeping the syringes in cold sterile water, when not in use.

**References.**

Landois, "Die Transfusion des Blutes," 1875.
Ottenberg, "Transfusion and Arterial Anastomosis," Annals of Surgery, April, 1908.
Crile, Tr. Am. Surg. Ass., Vol. XXVII.
Soresi, N. Y. Med. Journal, Nov. 9, 1912.
Cullen, Surg., Gyn., & Obstetrics, Sept., 1913.
Lespinasse, Journal A. M. A., June, 1914.
Bernheim, Journal A. M. A., July, 1913.
Ellsworth Eliot, N. Y. Surg. Soc., 1910.
Floercken, Muench. Med. Wochenschrift.
Ottenberg & Kaliski, Journal A. M. A., Dec. 1913.
Hennington, Buf. Med. Journal, Dec. 1913.
Landon, Journal A. M. A., August, 1913.
McGrath, Surg., Gyn., & Obstetrics, March, 1914. Journal A. M. A., Jan., 1914.
Janeway, Annals of Surg., May, 1911.
Brewer, Tr. Am. Surg. Ass., Vol. XXVII.
Pope, Journal A. M. A., April, 1913.
David & Curtis, Journal A. M. A., March, 1914.
Kimpton & Brown, Journal A. M. A., July, 1913.
Crotti, Surg., Gyn., & Obstetrics, Vol. XVIII.
Cooley & Vaughan, Journal A. M. A., Feb., 1913.
Lindeman, Am. Journal Dis. Children, July, 1913.
Editorial, Journal A. M. A., Aug., 1912.
Satterlee & Hooker, Surg., Gyn., & Obstetrics, Aug., 1914. Journal A. M. A., June, 1914.

519 E. Utica street.

---

**Chlorophyll Test for Gastric Motility.** Franklin W. White, Boston, Boston M. & S. Jour., Nov. 19, 1914. This is a conservative discussion of this colorimetric test which depends upon the algebraic estimation of residue from the dilution of a solution of known strength. As the author points out, it is open to the general fallacies of any similar test and especially to interference by bile (which shows green if HCI is present in any considerable amount) but it is somewhat simpler than certain analogous tests.

---

**Formula for Determining Surface Area of Infants.** J. Howland and R. T. Dana, Am. Jour. Dis. of Children, p. 33, 1913, vol. 6, criticise other formulae and claim the following to be quite accurate: Surface in square centimeters equals 0.483 times the weight plus 730.

---

**Quintuplets.** The Lancet of Aug. 22, 1914, cites the case of woman aged 25, delivered June 23 at the Malagamuda Orphanage, Zuculay, Travancore, India, all the foetuses being female.

